# *Giardia lamblia* infection increases risk of chronic gastrointestinal disorders

**DOI:** 10.1186/s40794-016-0030-0

**Published:** 2016-08-30

**Authors:** Megan Dormond, Ramiro L. Gutierrez, Chad K. Porter

**Affiliations:** 1 Enteric Disease Department, Naval Medical Research Center, 503 Robert Grant Avenue, Silver Spring, MD 20910 USA; 2 George Washington University, Washington, USA

**Keywords:** Functional gastrointestinal disorders, *Giardia lamblia*, Post infectious, Infectious gastroenteritis

## Abstract

**Background:**

*Giardia lamblia* is a common parasitic cause of infectious gastroenteritis in the United States and the world and may be linked to an increased risk of chronic gastrointestinal (GI) disorders. We sought to assess the risk of several chronic GI disorders following Giardia infection among active duty US military personnel.

**Methods:**

This study was designed as a retrospective cohort study in which active duty military personnel with documented *G. lamblia* infection were assessed for the subsequent risk of developing a chronic GI disorder including irritable bowel syndrome (IBS), dyspepsia and gastroesophageal reflux disease (GERD). Post-giardia chronic GI disorder risk was compared to risk in uninfected personnel matched on several demographic characteristics and medical encounter information. Data were obtained from the Defense Medical Surveillance System and exposures (1998–2009) with outcomes identified based on documented medical encounters with specific medical billing codes. Modified Poisson regression was used to evaluate the relationship between *G. lamblia* infection and chronic GI disorders.

**Results:**

A total of 80 Giardia cases were identified for an estimated incidence of 0.55 cases per 100,000 person-years. Cases were matched to 294 unexposed subjects. After adjusting for important covariates, there was an increased risk of IBS (relative risk: 2.1, *p* = 0.03) associated with antecedent Giardia infection.

**Conclusion:**

These data add to a growing body of literature and demonstrate an increased risk of IBS after infection with *G. lamblia.*

## Background

Infectious gastroenteritis (IGE) is caused by numerous bacterial, viral, and parasitic pathogens. Among parasites, *Giardia lamblia*, a unicellular eukaryotic microorganism that causes symptoms of diarrhea, abdominal cramps, and occasionally nausea and vomiting, is a predominate pathogen. Symptoms of giardiasis usually occur within 1–2 weeks following exposure; however, they can persist for weeks or even months without proper treatment [1]. Giardia accounts for approximately 77,000 cases of parasitic foodborne illness annually in the US [2] and is a known cause of travelers’ diarrhea among civilian and military travelers [3, 4]. Globally, the burden is even greater with recent literature from the Foodborne Disease Burden Epidemiology Reference Group (FERG) estimating 183 million cases and 171,000 disability adjusted life years (DALYs) attributed to *G. lamblia* in 2010 [5].

In addition to the acute illness, giardiasis has been associated with an increased risk of several secondary or chronic health conditions and is known to cause small intestinal malabsorption [1]. One such group of sequelae that has been increasingly associated with other enteric pathogens are functional gastrointestinal disorders (FGD). The pathophysiology of FGDs is likely varied and has yet to be clearly characterized but is typified by the onset of chronic or recurrent GI symptoms [6]. The FGDs have been categorized into 24 separate diagnoses based on the “Rome Diagnostic Criteria” [7, 8]. As a group, these disorders are estimated to account for more than 11 million ambulatory care visits, equating to roughly four visits for every 100 persons in the United States [8]. The pathoetiology of these disorders is poorly understood; however, several mechanisms have been implicated in producing symptoms including gut brain axis dysfunction, mucosal barrier disruption, gastrointestinal dysmotility, microbiota disturbances, inflammation, visceral hypersensitivity, diet, and genetic predisposition [8].

We sought to assess the association between *G. lamblia* infection and several FGD in active duty US military personnel, a healthy subset of the general travel population*.* The outcomes studied have been previously shown to be associated with infectious gastroenteritis [8–12]; however, data linking antecedent Giardia infections are lacking.

## Methods

This retrospective cohort study evaluated the risk of PI sequelae following a documented parasitic IGE episode compared to the risk of those same outcomes in a cohort of unexposed subjects. Exposed subjects were identified by the International Classification of Diseases, volume 9 – Clinical Modification (ICD9-CM) codes using the Defense Medical Surveillance System (DMSS). Giardia exposure was identified based on a documented medical encounter with 007.1 visit code. Up to four unexposed subjects were matched to each Giardia case based on an unrelated medical encounter within 1 year of the Giardia exposure exposed. Unrelated medical encounters codes included the following: acute respiratory infections (ICD9-CM: 460–466), pneumonia and influenza (ICD9-CM: 480–488), infectious of the skin and subcutaneous tissue (ICD9-CM: 680–686), dislocations (ICD9-CM: 830–839), sprains and strains for joints and adjacent muscles (ICD9-CM: 840–848), burns (ICD9-CM: 910–949), fracture of upper or lower limb (ICD9-CM: 810–829). Additional matching criteria included age (±1 year), gender, number of prior deployments, medical treatment facility and type of medical encounter (inpatient or outpatient). All subjects had a minimum of 1 year of documented follow-up within the DMSS and were selected from the total active duty US military population from 1998 to 2009. The outcomes of interest were irritable bowel syndrome (IBS) (ICD9-CM: 564.1, 306.4), constipation (ICD9-CM: 564.0), dyspepsia (ICD9-CM: 536.8) and gastroesophageal reflux disease (GERD) (ICD9-CM: 530.81).

Additional covariates were included to determine their effect on the risk of PI sequelae. ICD9-CM codes for Axis I psychological disorders of interest included neurotic disorders (300), personality disorders (301), sexual disorders (302), acute reaction to stress (308), adjustment reaction (309), non-psychotic brain syndrome (310), depressive disorders not elsewhere classified (311), conduct disturbances not elsewhere classified (312), emotional disturbance (313), and hyperkinetic syndrome (314).

Incidence of Giardia was estimated based on the number of cases identified and the total active duty military population data obtained from the Defense Manpower Data Center. Descriptive characteristics were calculated and stratified by exposure. Associations between the exposure and outcomes were initially explored using appropriate univariate methods. Modified Poisson regression models using a robust sandwich estimator for variance were used to investigate the relationship between giardiasis and each of the outcomes independently [13]. Multivariate models were built for each outcome using backwards elimination and an *alpha* = 0.15 to retain variables. All statistical analyses were conducted using SAS v. 9.3 for Windows (SAS Institute, Cary, North Carolina).

## Results

A total of 80 giardiasis cases were identified for an estimated incidence of 0.55 per 100,000 person-years in males (no female cases were identified). Giardiasis cases were matched to 294 unexposed subjects for a total sample size of 374 subjects. All of subjects were male with a median age of 35 years (interquartile range: 32, 46) at the time of study completion (Table 1). Over two-thirds (*n* = 272) of the study population had at least a high school education while the remaining (*n* = 65) had either bachelor’s (*n* = 31, 8.2 %) or master’s degrees (*n* = 34, 11.6 %) (Table 1). Occupation was fairly evenly distributed among all the possible categories. In terms of branch of service, many of the study subjects were in the Navy (31.1 %) followed closely by the Army (29.4 %) and finally the Marines (22.2 %) (Table 1).

**Table 1:** Demographic characteristics of selected US military members between 1998–2009

	*G. lamblia*	Unexposed	Total
N	80	294	374
Age [Median (IQR)]	37.35 (34, 45)	35 (30, 46)	35 (32, 46)
Sex [n (%)]			
Male	80 (100)	294 (100)	374 (100)
Race/Ethnicity [n (%)]			
Hispanic	13 (16.3)	69 (23.5)	82 (23.7)
White	67 (83.8)	215 (73.1)	282 (75.4)
Other/Unknown	0 (0)	10 (3.4)	10 (2.6)
Education [n (%)]			
High School	55 (68.8)	217 (73.8)	272 (72.7)
Some College	10 (12.5)	25 (8.5)	35 (9.4)
College	5 (6.3)	26 (8.8)	31 (8.2)
Graduate School	10 (12.5)	24 (8.2)	34 (11.6)
Unknown	0 (0)	1 (0.3)	1 (0.3)
Rank [n (%)]			
Junior enlisted	23 (28.8)	116 (39.5)	139 (50.7)
Senior enlisted	44 (55.0)	130 (44.2)	174 (46.5)
Junior officer	0 (0)	2 (0.7)	5 (0.8)
Senior officer	13 (16.3)	46 (15.6)	59 (15.7)
Service [n (%)]			
Army	29 (36.3)	81 (27.5)	110 (29.4)
Coast Guard	0 (0)	12 (4.1)	12 (3.2)
Air force	12 (15)	41 (13.9)	53 (14.2)
Marines	13 (16.3)	70 (23.8)	83 (22.2)
Navy	26 (32.5)	90 (30.6)	116 (31.1)
Military Operation [n (%)]			
Iraqi Freedom (Iraq)	17 (21.24)	85 (28.9)	102 (76.7)
Enduring Freedom (Afghanistan)	8 (10.0)	49 (16.6)	57 (65.4)
Primary Outcomes			
Dyspepsia	8 (10.0)	5 (17)	13 (3.5)
Constipation	4 (5.0)	6 (20.4)	10 (2.6)
IBS	16 (20.0)	16 (5.4)	32 (8.5)
GERD	26 (32.5)	51 (17.3)	77 (20.5)

In univariate analyses (Table 2), *G. lamblia* infection was associated with an increased risk of IBS (relative risk {RR} [10]: 2.1; 95 % confidence interval {CI}: 1.1, 3.7) and dyspepsia (RR: 3.2; 95 % CI: 1.2, 8.9) compared to unexposed subjects (Table 2). Those individuals that were diagnosed with an Axis I disorder were also at a 6.7 times (95 % CI: 3.7, 12.2) increased risk for IBS. GERD (4.0, 95 % CI: 2.9, 5.6), and dyspepsia (RR: 5.4, 95 % CI: 1.7, 16.5) were also significantly associated with Axis I disorders. Other bacterial and viral IGE episodes were associated with the development of IBS (RR: 5.9, 95 % CI: 3.2, 10.8), GERD (RR: 2.1, 95 % CI: 1.3, 3.5) and functional constipation (RR: 9.0, 95 % CI: 2.7, 29.8).

**Table 2:** Unadjusted incident rate ratios (95 % confidence intervals) for outcomes of interest and selected covariates evaluating risk of functional gastrointestinal disorders following acute *G. lamblia* infection

Covariate	Outcome of interest			
	IBS	GERD	Dyspepsia	Constipation
Non-Caucasian	0.5 (0.2, 1.4)	1.2 (0.8, 1.8)	0.9 (0.3, 3.0)	0.4 (0.1, 2.9)
> High school education	1.5 (0.8, 3.0)	0.9 (0.6, 1.3)	0.8 (0.2, 2.7)	1.7 (0.5, 5.7)
Officer	1.6 (0.7, 3.3)	1.3 (0.9, 2.0)	1.8 (0.6, 5.1)	5.4 (0.7, 43.5)
Non-Army Service	1.8 (0.7, 4.4)	1.5 (1.0, 2.4)	2.3 (0.6, 9.8)	1.7 (0.4, 7.6)
Operational deployment	2.0 (0.7, 5.6)	1.0 (0.5, 2.2)	0.4 (0.1, 3.3)	0.7 (0.1, 7.6)
Axis I disorder	6.7 (3.7, 12.2)	4.0 (2.9, 5.6)	5.4 (1.7, 16.5)	18.4 (4.2, 81.1)
Bacterial or Viral Infection	5.9 (3.2, 10.9)	2.1 (1.3, 3.5)	3.8 (1.0, 15.2)	9.0 (2.7, 29.8)
*G. lamblia*	2.1 (1.1, 3.7)	1.1 (0.7, 1.6)	3.2 (1.2, 8.9)	1.3 (0.4, 4.8)

In multivariate models (Table 3), the risk of all outcomes remained elevated in those with Axis I disorders with adjusted RR {aRR} ranging from 3.4 to 12.5. Individual disorders were not investigated independently due to low numbers of each diagnosis. In contrast, military rank and race appeared to have differential effects across the outcomes of interest with officers and non-Caucasian subjects with a decreased risk of IBS, but an increased risk of functional constipation. After adjustment for covariates (Table 3), only IBS retained a significant association with giardiasis (aRR: 2.1, 95 % CI: 1.1, 4.0).

**Table 3:** Adjusted incident rate ratios (95 % confidence intervals) for outcomes of interest and selected covariates evaluating risk of functional gastrointestinal disorders following acute *G. lamblia* infection

	IBS	GERD	Dyspepsia	Constipation
Officer	0.3 (0.1, 1.0)	1.7 (0.7, 3.9)	1.2 (0.2, 7.8)	6.8 (2.0, 22.8)
Non-Caucasian	0.3 (0.1, 1.0)	1.9 (0.8, 4.3)	1.2 (0.2, 9.7)	2.8 (0.6, 13.2)
Axis I disorder	12.5 (4.1, 37.7)	3.4 (2.4, 4.7)	4.6 (1.3, 16.5)	9.8 (2.2, 43.7)
Bacterial or Viral Infection	2.3 (0.8, 6.4)	1.3 (0.7, 2.4)	1.8 (0.4, 8.2)	2.7 (0.7, 9.2)
*G. lamblia*	2.1 (1.1, 4.0)	1.0 (0.7, 1.4)	2.6 (0.9, 7.4)	1.2 (0.3, 5.2)

## Discussion

This study demonstrated over a 2-fold risk of IBS among those with an antecedent Giardia infection and an unexposed reference population after adjusting for covariates. While several studies have described the incidence of FGD following bacterial and viral IGE few have examined their incidence subsequent to parasitic infection [8, 14].

The incidence of Giardia reported here is lower than recorded in another recently published study in this population. Specifically, the Medical Surveillance Monthly Report (MSMR) published in October of 2013 cites an 11-year surveillance period of the US Armed Forces for gastrointestinal infections [15]. The authors reported an incidence rate of giardiasis of 6.2 per 100,000. The study reported here utilized data from 1998 to 2009, while the MSMR focused on 2002–2012 [15]. The highest rates of Giardia from the MSMR study were in 2002–2006 and from 2008 to 2012 [15]. Case diagnosis year was not available, precluding annual comparisons across the two studies. One potential reason for the difference in incidence estimates could be variable inclusion criteria. For inclusion in this study, a subject could not have a prior documented medical encounter with any of the outcomes of interest (IBS, GERD, constipation, dyspepsia) to help cut down on the likelihood that the outcome of interest could be attributed to something other than the parasitic exposure. If a participant had a prior medical encounter with one of the outcomes of interest it would underestimate the incidence.

The results of this study are consistent with other studies reporting an increase in IBS after enteric infection [16–18]. While not specific to parasitic infections, two recent meta-analyses have shown that the risk increases 6- to 7-fold after IGE and remains elevated for at least 2 to 3 years after the initial infection [9, 10]. In only one prior study has antecedent *Giardia* infection been linked to IBS. Following a Giardia outbreak in Bergen, Norway, investigators found a 46.1 % IBS prevalence in exposed subjects (46.1 %) compared to 14.0 % in the control subjects (aRR: 3.4, 95 % CI: 2.9, 3.8) [17]. Similarly, we found a 2.1 increase risk in IBS following sporadic Giardia infections compared to an uninfected reference population. The estimate reported here (aRR: 2.1) is lower than that reported in prior systematic reviews or in the prior *G. lamblia* outbreak possibly due to case definitions, study design and/or differences in study populations. The Bergen study used a questionnaire mailed to participants with standardized questions where IBS was defined according to the Rome III criteria, whereas we relied on passive surveillance through DoD medical encounters [17].

Evidence of the association between acute IGE and development of functional dyspepsia has been accumulating in the literature over the past decade. The findings of this study support the growing body of evidence demonstrating an association between acute GI infection and functional dyspepsia [16, 18]. FGD was the third most prevalent FGD found within the study population (overall estimate of 3.5 %); however, this is probably an underestimate. Most studies show the prevalence of post-infectious FD around 10 % [11]. Quigley and Lacy reported a prevalence of 12–15 % among the general US population [19]. A meta-analysis in 2013 estimated a pooled OR of 2.18 (95 % CI: 1.70, 2.81) for the risk of developing FD following IGE [12]. Dizdar recently reported that patients with abdominal symptoms after acute *Giardia* infection had evidence of enhanced visceral sensitivity, and 15 of the 22 patients had evidence of FD indicating that dyspepsia may be attributed to increased visceral sensitivity that persists after initial infection [16]. A recent study [16].

This study found a similar trend with more cases having a GERD diagnosis (32.5 %) compared to controls (17.3 %); however, this was not significant in multivariate analyses. A 2012 study looking at three recent norovirus outbreaks found the risk of GERD is higher among those who had an IGE diagnosis during a confirmed outbreak (aRR: 1.39, 95 % CI: 1.07, 1.81) [20]. The potential association between Giardia and functional dyspepsia needs further study.

The mechanisms by which Giardia infection may induce chronic, long-term FGD are unclear. Recently, researchers at the University of Calgary have shown that infection with Giardia can induce functional changes in commensal flora composition in a nematode model [21]. In a healthy host, it may be that these infections trigger the intestinal dysbiosis and altered sensory perception common noted in many FGDs [22]. Additionally, Giardia has been shown to disrupt tight junctions, which perhaps alters gut permeability in a way that alters normal homeostasis and host-microbiota interactions [23]. The link between giardiasis and post-infectious sequelae is likely the result of a multi-factorial process in a susceptible host and understanding these processes and host characteristics is an ongoing area of research across multiple institutions.

Psychological comorbidities, such as stress and anxiety, are associated with an increased IBS risk, and often the two conditions occur together [14, 24, 25]. In fact, we observed a stronger association between axis I disorders and FGDs then was observed with our exposure of interest (ie, Giardia infection). Several studies have demonstrated a similar association between axis I disorders and IBS [8, 9, 24, 25]. Additionally, traumatic life events can often precede the onset of functional bowel disorders [26]. A meta-analysis conducted in 2002 found that psychiatric comorbidities were present in up to 94 % of patients with chronic gastrointestinal disorders, including IBS [27]. We observed an increase in the risk of IBS, GERD, dyspepsia, and constipation in subjects with preceding axis I diagnoses after controlling for relevant covariates and exposures. These results are consistent with observed associations between psychological co-morbidities and IBS [9, 10]. It is important to note, that the associations between giardia and chronic GI disorders were noted to be significant when controlling for these important known covariates. Interactions between IGE and psychological co morbidities were difficult to investigate due to low numbers of each individual comorbidity.

Limitations inherent in our study may impact the generalizability of these results. Giardia infections were identified exclusively among males in this study population, perhaps not unsurprising given the study population and the inclusion criteria. Interactions between covariates were not investigated due to a small sample size of the outcome of interest and relatively low numbers of participants within each subcategory of covariates. FGD diagnoses are complicated by their functional nature and the lack of diagnostic tests [7]. It is also possible that the observed findings could be the result of an unmeasured confounder; however, no confounders have been proposed to date that would differentially affect the exposed cohort. The etiology of enteric infection is also complicated by poor diagnostic test utilization and a low rate of care-seeking behavior in persons with IGE. The utilization of ICD9-CM codes to identify exposures and outcomes can be problematic and has been described elsewhere [28].

## Conclusions

Despite the limitations, this study suggests an increased risk of chronic GI outcomes following Giardiasis similar to what has been reported with other enteric infections. Given recent literature from the FERG on the *G. lamblia*-specific global morbidity [5] and the fact that Giardia is a major contributor to food and waterborne illnesses in the United States [2], understanding the association between acute infection and these chronic sequelae is important and will yield more accurate estimates of global disease morbidity.

## Abbreviations

FGD: Functional gastrointestinal disorder
GERD: Gastroesophageal reflux disease
GI: Gastrointestinal
IBS: Irritable bowl syndrome
